# Antimony Removal from Water by a Chitosan-Iron(III)[ChiFer(III)] Biocomposite

**DOI:** 10.3390/polym11020351

**Published:** 2019-02-18

**Authors:** Byron Lapo, Hary Demey, Tanya Carchi, Ana María Sastre

**Affiliations:** 1 School of Chemical Engineering, Universidad Técnica de Machala, UACQS, BIOeng, 070151 Machala, Ecuador; tanyacarchi@gmail.com; 2 Department of Chemical Engineering, Universitat Politècnica de Catalunya, ETSEIB, Diagonal 647, 08028 Barcelona, Spain; ana.maria.sastre@upc.edu; 3 Commissariat à l’Energie Atomique et aux Energies Alternatives, CEA/DRT/LITEN/DTBH/STBH/L2CS, 17 rue des Martyrs, 38054 Grenoble, France

**Keywords:** antimony removal, chitosan, iron, sorption

## Abstract

The presence of antimony(III) in water represents a worldwide concern, mainly due to its high toxicity and carcinogenicity potential. It can be separated from water by the use of sustainable biopolymers such as chitosan or its derivatives. The present study applied chitosan modified with iron(III) beads to Sb(III) removal from aqueous solutions. The resulting material performed with a high adsorption capacity of 98.68 mg/g. Material characterization consisted of Raman spectroscopy (RS), X-ray diffraction (XRD), scanning electron microscope observations (SEM-EDX), Fourier transform infrared spectroscopy (FTIR) and point of zero charge (pH_pzc_). The adsorption study included pH study, effect of initial concentration, kinetics, ion effect, and reusability assessment. The RS, XRD, and FTIR results indicated that the main functional groups in the composite were related to hydroxyl and amino groups, and iron oxyhydroxide species of α-FeO(OH). The pH_pzc_ was found to be 7.41. The best adsorption efficiency was set at pH 6. The equilibrium isotherms were better fitted with a non-linear Langmuir model, and the kinetics data were fitted with a pseudo-second order rate equation. The incorporation of iron into the chitosan matrix improved the Sb(III) uptake by 47.9%, compared with neat chitosan (CS). The material did not exhibit an impact in its performance in the presence of other ions, and it could be reused for up to three adsorption–desorption cycles.

## 1. Introduction

The worldwide effects of heavy metals have awakened interest for finding suitable methods to remove contaminants from water sources. Among the toxic heavy metals, antimony is one of the most lethal elements; however, few investigations have been carried out to mitigate its presence in aqueous solutions. Antimony generally exists in two oxidation states: Sb(III) and Sb(V), of which Sb(III) is 10 times more toxic than Sb(V) [1]. It can reach the environment mainly through the mining and processing of antimony-containing ores, industries related to antimony based products [2,3] and it can be found in drinking water from PET bottles [4]. 

According to the World Health Organization (WHO), the recommended limit concentration of antimony set in the guidelines for drinking water as 20 µg/L; its occurrence in groundwater is less than 0.001 µg/L; meanwhile, in surface water, it is less than 0.2 µg/L and less than 5 µg/L in drinking-water [5]. The removal of this element from water sources can be carried out by several methods such as coagulation-flocculation [6], electrocoagulation [7], membrane filtration [8], adsorption [9], etc. Between them, sorption (particularly bio-sorption) represents a sustainable alternative, due to its low cost and the abundance of the materials used (renewable). Among the biomaterials that are commonly used in the literature can be mentioned: chitosan, alginates, cellulosic and cell biomass, which have been tested in the raw state, or as support matrices for the manufacturing of innovative sorbents [10,11,12]. 

Chitosan biopolymers represents the second omnipresent bioresource in the world (after cellulose), it is an efficient biosorbent to be used directly or modified chemically, for the extraction of the target element. To separate Sb(III) from water, chitosan particles have been modified in several configurations, such as nano-titania-crosslinked chitosan [13], chitosan-modified pumice [14], etc. On the other hand, iron in various forms (iron oxides, binary metal iron oxides, soil enriched with ferric ion and zero-valent iron, and iron-loaded composites) have been demonstrated to be good sorbents for the removal of Sb [15]; e.g., Xu et al. [16] used iron-based materials to efficiently separate Sb(III) from water, and the high adsorption capacity obtained can be compared with other inorganic materials such as bentonite, manganite, or goethite.

Based on the precept that this hybrid composite is made from renewable, low-cost, and natural waste biomass, our research group has developed a simple material based on chitosan-Fe(III), labeled ChiFer(III), which was previously reported to have excellent performance in the removal of boron, mercury, lead, and neodymium ions [17,18,19]. Thus, the present research is focused on assessing the technical feasibility of separating Sb(III) from water, through the use of ChiFer(III) beads; the incorporation of iron(III) into the chitosan matrix resulted in an enhancement of the uptake capacity towards antimony, and the material showed excellent stability in terms of ion competition and reusability.

## 2. Materials and Methods

### 2.1. Chemicals

All chemical reagents used in this work were of analytical grade, and the solutions were prepared with deionized water type II. A stock solution of 1000 mg Sb(III)/L was prepared from KSbC_4_H_4_O_7_ (99.95% Probus, Barcelona, Spain), the neat chitosan with an average molecular weight of 125,000 g/L and a degree of acetylation of 0.13 (Aber Technologies, Lannilis, France) previously reported [20], acetic acid (CH_3_COOH, 99.7%, Panreac, Barcelona, Spain), sodium borohydride (NaBH_4_, ≥96%, Sigma-Aldrich, St. Louis, MO, USA), nitric acid (HNO_3_, 69.0%, J.T. Baker, Radnor, USA) sodium chloride (NaCl, 99.5%, Prolabo, Fontenay-sous-Bois, France), and iron(III) chloride (FeCl_3_·6H_2_O, 99%, Fluka, Buchs, Switzerland).

### 2.2. Preparation of Composite Beads

ChiFer(III) beads were prepared according to Demey et al. [19]. Briefly, a chitosan solution with a concentration of 2.2% (*w*/*w*) was prepared (solution A): 30 g chitosan was dissolved in 2.2% (*w*/*w*) acetic acid solution (1350 mL) and stirred for 5 h. Simultaneously, a solution B consisted in iron(III) was prepared by mixing 30 g FeCl_3_·6H_2_O in 120 mL of 0.5 M HCl solution (until complete dissolution). 

Then, both A and B solutions were mixed under vigorous stirring (500 rpm) for 2 h. The chitosan–iron(III) mixture was added drop-by-drop with a peristaltic pump through a thin nozzle (Ø 2 mm) into an agitated bath of 1 M NaOH for producing the spheres of ChiFer(III) material. The formed beads were kept under stirring for 8 h, and then washed with distilled water (until neutral pH 7) prior to freeze-drying (by a LyoQuest-55, Telstar equipment, São Paulo, Brazil). Moreover, raw neat chitosan (CS) was used to evaluate the performance differences with ChiFer(III) material. 

### 2.3. Characterization

Raman spectra were obtained from 4000 to 150 cm^−1^, using a Renishaw microscope (Renishaw in via microscope, Wotton under Edge, UK) with Nd:YAG laser excitation and 2 cm^−1^ resolution, Fourier transform infrared spectroscopy (FTIR) was performed in a Thermo Nicolet spectrometer (Thermo Scientific, Waltham, MA, USA). X-ray diffraction (XRD) patterns were obtained in a Bruker D8 Advance (Bruker AXS GmBH, Karlsruhe, Germany) spectrometer, using Cu Kα radiation collected in 4–100° 2 theta, with a scanning rate of 0.02°/s. The point of zero charge (pH_pzc_) was obtained following the methodology of Yazdani et al. [18] in a Mettler Toledo, SevenMulti pH meter (Tiel, The Netherlands); the so-called ‘drift method’.

### 2.4. pH Study

For determining the optimum pH in which the ChiFer(III) material reach the highest performance, several desorption tests were carried out at different pHs (from pH 2 to 6). A known volume of solution (25 mL) with an initial metal concentration of 50 mg/L was mixed with 25 mg of dried beads (sorbent dosage, S.D., of 1 g/L), and stirred for 24 h (150 rpm) for achieving the equilibrium. At the end of the experiments, the final pH was measured, and 5 mL of solution was filtered and analyzed with hydride vapor generation coupled to the atomic absorption technique (HVG-AA method: Shimadzu AA6300 spectrophotometer, Shimadzu Corp. Kyoto, Japan). The desorption capacity (q_e_) versus pH was reported. 

### 2.5. Effect of Initial Concentration

The desorption isotherms were obtained by using different initial metal concentrations (from 30 to 400 mg/L). A known volume of solution (25 mL) at pH 6, was mixed with 25 mg of ChiFer(III) and agitated on an orbital shaker for 24 h (at 150 rpm). After that, the remaining Sb(III) concentration was measured by the hydride vapor generator coupled to atomic absorption spectrophotometry (HVG-AA) technique. The experimental isotherms were built by plotting the desorption capacity (q_e_) versus the equilibrium concentration (C_e_). The experimental data were fitted with the non-linear models of Langmuir and Freundlich, according to Equations (1) and (2).

The non-linear Langmuir model [21]:

(1) $$q_{e}=\frac{q_{\max}{\mathrm{bC}}_{e}}{1+{\mathrm{bC}}_{e}}$$

where q_e_ is the amount of metal adsorbed in (mg/g), C_o_ and C_e_ are the initial and equilibrium concentrations respectively in (mg/L), q_max_ is the Langmuir adsorption maximum capacity expressed in (mg/g), and b is the Langmuir constant in (L/mg).

The non-linear Freundlich model [22] is as follows:

(2) $$q_{e}=K_{F}C_{e}^{1/n}$$

where q_e_ is the amount of metal adsorbed per mass of sorbent (mg/g), C_e_ is the equilibrium concentration, K_F_ is the Freundlich constant, and n is sorption intensity.

### 2.6. Kinetics

The uptake experiments were performed by adding a known amount of sorbent (S.D. 1 g/L) to 1 L of antimony solution (100 mg/L) at pH 6, Aliquots of solutions were withdrawn at different times over 48 h of contact time, with a chitosan–iron(III) material. The residual concentration was determined by the HVG-AA technique. The models, such as pseudo-first- and pseudo-second-order rate equations (PFORE and PSORE, respectively) were used for fitting the experimental data.

The pseudo-first-order rate equation (PFORE) [23] is as follows:

(3) $$\frac{{\mathrm{dq}}_{t}}{\mathrm{dt}}=K_{1}\left(q_{\mathrm{eq}}-q_{t}\right)$$

The pseudo-second-order rate equation (PSORE) [24] is as follows:

(4) $$\frac{{\mathrm{dq}}_{t}}{{\left(q_{\mathrm{eq}}-q_{t}\right)}^{2}}=K_{2}\mathrm{dt}$$

where q_eq_ is the equilibrium sorption capacity (mg/g), q_t_ is the sorption capacity (mg/g) at any time t (min) and K_1_ (1/min) and K_2_ (g/mg min) are the pseudo-first- and pseudo-second-order rate constants, respectively. 

### 2.7. Ion Competition

In order to evaluate the interaction (or competition) of the common ions that coexist with antimony in the real systems, synthetic wastewater was prepared and put into contact with the sorbent material for 24 h at 150 rpm. Two batch experiments were carried out, with Sb(III) solutions of 10 mg/L and 100 mg/L at pH 6, being mixed with Na_2_NO_3_: 0.15 mM, NaCl:1 mM, Na_2_CO_3_: 2 mM Na_2_SO_4_: 2 mM. 

### 2.8. Reusability Assessment

Adsorption–desorption cycles were carried out in order to determine the possibility of reuse of the material. HCl pH 3.5 and ethylenediaminetetraacetic acid (EDTA) pH 10 eluents were assessed to test the better desorbing solution. Three adsorption–desorption cycles were performed; the desorption efficiency was calculated according to Equation (5). All tests were made in duplicate.

(5) $$\%\;\mathrm{desorption}=\frac{m_{A}-m_{D}}{m_{A}}*100$$

where m_A_ and m_D_ are the sorbed and eluted mass of the metals (mg) at each adsorption/desorption cycle, respectively.

## 3. Results and Discussion

### 3.1. Characterization of the Material

#### Raman, XRD Spectroscopy, and SEM Observations

**Figure 1:**
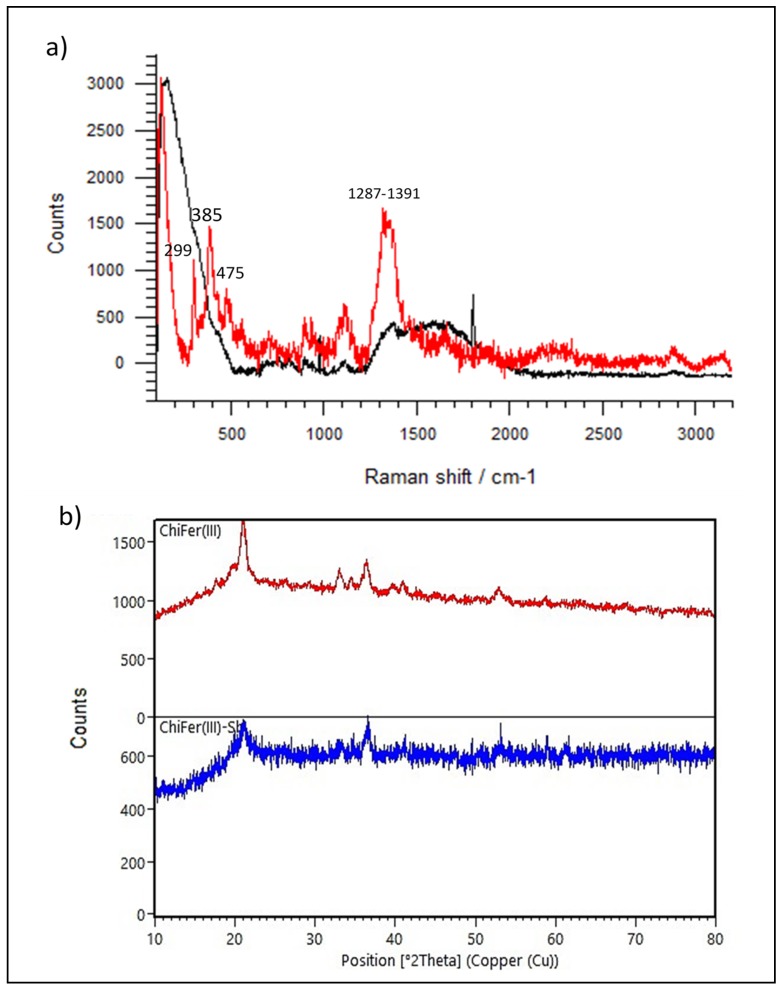
Raman (RS) and X ray diffraction (XRD) spectra. (**a**) Black line: neat CS raman spectra, red line: ChiFer(III) raman spectra. (**b**) Red line: ChiFer(III) XRD spectra, blue line: ChiFer(III) Sb-loaded XRD spectra.

Raman spectroscopy is a useful technique for obtaining information about the molecular and electronic structure of the material surface. Figure 1a shows the RS spectra of the sorbent material, where it is possible to differentiate the functional groups of the neat chitosan and ChiFer(III). Also, in Supplementary Materials, Figures S1–S3, the IR spectra of CS and ChiFer(III) before and after Sb(III) adsorption, respectively, are presented.

According to the RS of neat chitosan, in Figure 1a, a broad band between 1287–1391 cm^−1^ was observed, which involved δ(CH_2_), δ(CH), δ(OH), and those related to v(C–O–C), v(C–OH), v(C–CH2), δ(CH), p(CH2), and p(CH)3, in the range of 1089–1115 cm^−1^. Also, it was noticed that the peak at 1591 cm^−1^ corresponded to δ(NH_2_) vibration, i.e., this was related to the partial acetylation of the NH_2_ group of chitosan [25]. In ChiFer(III), the broad band between 1287 to 1391 cm^−1^ showed an increase in its intensity, probably because the related functional groups were occupied by iron(III). Moreover a strong intensity band appeared at 385 cm^−1^, 299 cm^−1^, and 475 cm^−1^, which could be related to a goethite form of iron [26]. On the other hand, the bands at 349 cm^−1^ and 443 cm^−1^ of δ(OH); 563, 1094, corresponding to δ(NH), δ(C=O), α(CH3), and 1683 cm^−1^ were related to stretching vibrations of v(CO), and disappeared in ChiFer(III), and were probably occupied by iron ions.

Furthermore, the FTIR spectra of CS (Supplementary Materials, Figure S1), showed a broad characteristic peak in the region of 3100–3600 cm^−1^, relating to the –OH and NH_2_ stretching vibrations, which were less pronounced in the ChiFer(III) material, and probably related to the formation of Fe-OH (Figure S2). The peak at 2362.53 cm^−1^ appeared in ChiFer(III) and shifted to 2360.0 cm^−1^ after Sb(III) adsorption, which is related to the incorporation of iron on the CS (Figure S3). According to [27] the stretching vibration at 1629 cm^−1^ and 1507 cm^−1^ were related to α-FeOOH, which appears in ChiFer(III) and disappears after Sb(III) adsorption.

The XRD patterns of ChiFer(III) were acquired and are shown in Figure 1b; it was found that the characteristic peaks of orthorhombic α-FeOOH were as follows: (21.02), (33.06), (34.51), (36.43), (40.96), (52.90) and (58.77) [28]; furthermore, the pattern matched with XRD data from the crystallography open database (COD) file: 96-900-3077. These findings are in concordance with the results obtained from FTIR and Raman spectroscopy, and they confirm the existence of goethite in the chitosan. After antimony adsorption, ChiFer(III) presented some differences in the pattern, and the peaks at (21.17), (33.06), and (36.69) remained similar; however, the peaks at (34.51), (36.43), (40.96), and (52.90) disappeared, while some peaks appeared: (53.06), (58.98), and (61.32), probably due to the interaction between the composite and Sb on the ChiFer(III) surface. According to [29,30], it is probable that the oxidation of Sb(III) into Sb(V) due to the presence of O_2_, could be a result of this these phenomena. 

**Figure 2:**
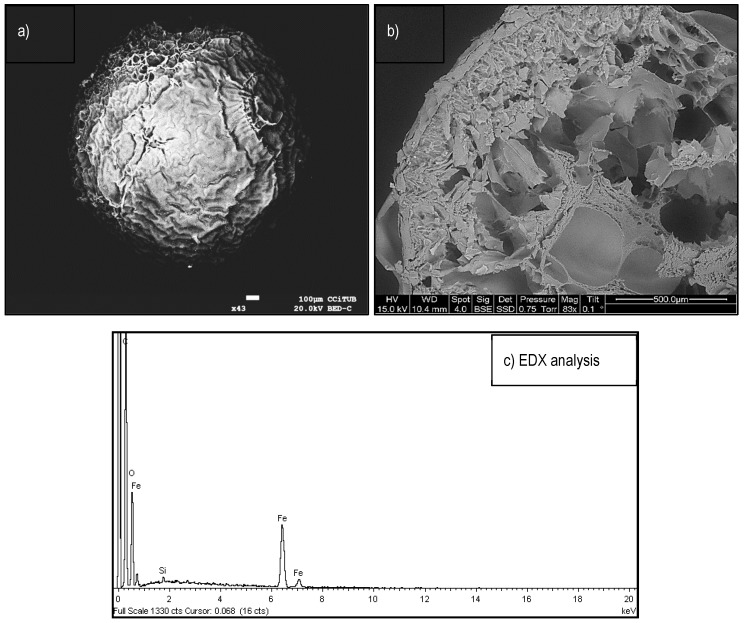
Scanning electron microscopy (SEM) observations. (**a**) Bead photo of ChiFer(III). (**b**) ChiFer(III) segment. (**c**) Energy-dispersive x-ray (EDX) spectra.

In order to observe the form and morphology of the material, an scanning electron microscopy and energy-dispersive X-ray (SEM-EDX) analysis is presented in Figure 2.

In Figure 2a, is possible to observe the spherical forms and roughness surfaces of the beads. The average particle size (ps) was 0.5 mm (0.1 < ps < 1.05 mm), which was confirmed by light scattering measurements (Figure S7). Alternatively, Figure 2b shows a bead segment (cross-section) where the inner morphology can be observed, which present high degrees of roughness and porosity in different pore sizes (25–300 µm). This configuration probably favors liquid transport, and consequently, it can facilitate the mass transfer of ionic Sb species throughout the sorbent material. To complement the SEM observations, Figure 2c showed the elemental composition of the bead where the presence of iron in the composite was previously described by RS, XRD and FTIR analyses.

### 3.2. Effect of the pH

**Figure 3:**
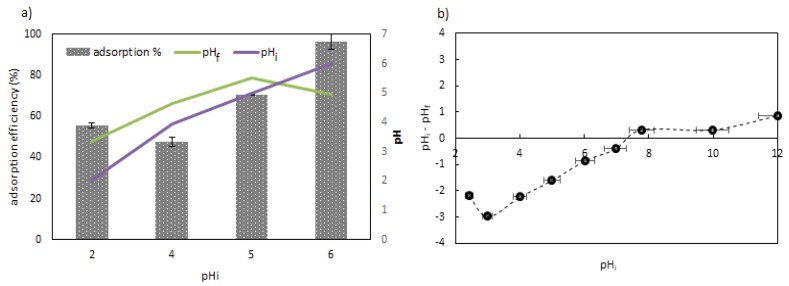
Effect of pH on antimony adsorption from aqueous solutions. (**a**) Performance of ChiFer(III) at different pHs (T = 20 °C; C_o_: 50 mg/L; dose: 1 g/L; contact time: 24 h; agitation rate: 150 rpm), (**b**) pH_pzc_ of ChiFer(III) (T = 20 °C; Electrolite: NaCl 0.01 M; dose: 1 g/L; contact time: 24 h; agitation rate: 150 rpm). pH_i_ = initial pH; pH_f_ = final pH.

Figure 3 presents the pH dependence in the adsorption of Sb(III) ions, as well as the potential of the zero charge (pH_pzc_) of the material. 

The influence of pH is one of the most important parameters to be assessed in the adsorption process, since it contributes to the optimization of the best operation conditions. Figure 3a shows that that the best sorption pH occurred at pH = 6, with around 96.62% of Sb(III) removal from an initial concentration of 50 mg/L, while at pH = 5, it skirted 70.52%, being even less at pH = 4, with around half of the best performance at pH = 6. Besides, from the error bars, a low variability between the data was found (RSD < 4.42%). The adsorption in the aqueous solutions had a strong pH dependence, mainly due to the characteristics of the sorbent material and the chemical species presented. The antimony species in aqueous solutions were $\mathrm{Sb}{\left(\mathrm{OH}\right)}_{2}^{+}$ (pH < 2) and the neutral complex of Sb(OH)_3_ (aq) (1.3 < pH < 12) [31,32,33]. These species interacted with the functional groups of the ChiFer(III) material reported in the FTIR (Supplementary Materials, Figure S2) and RS analyses (Figure 1a), which are mainly formed by amino-, OH-, and C-related groups, and iron metal ions. Moreover, in Figure 3a, where the pH change is represented by a secondary “y” axis; under pH 5, a positive variation in pH was noticed, and the change was less pronounced as the initial pH was incremented; this was probably due to the protonation of the ChiFer(III) surface. However, at an initial pH of 6, a decrease in the final pH after adsorption was observed; this occurred by the buffer effect of the chitosan; consequently, at this pH, the maximum adsorption performance was achieved.

On the other hand, the pH_pzc_ of the sorbent material was 7.41 (obtained graphically from Figure 3b). The pH_pzc_ represented the pH point where the majority of the surface sites were neutral [34]; this means that at pH < pH_pzc_, the surfaces of the sorbents were positively charged, and consequently, the removal of cation species decreases at an acidic pH, since the repulsion forces make it difficult for ions to diffuse onto the active sites of the sorbent. Naturally, by increasing the pH, the surfaces become more negative, and the sorbate/sorbent attractions improve the adsorption uptake of antimony [35,36]. It could also be interpreted that with the competition for active sites between the H^+^ and the Sb(III) species at acidic pH, thus, with an increase in pH, the adsorption toward Sb(III) is more favorable.

### 3.3. Effect of the Initial Concentration

The effect of the initial concentration on the performance of sorbent material is necessary for the construction of the adsorption isotherms. An adsorption isotherm can describe the phenomena governing the retention, release, or mobility of a substance from the aquatic environment to a solid sorbent, at a constant temperature and pH [37], as well as to adjust the data for mathematical models. The fit of the data by adsorption models is crucial for describing the distribution of the sorbate between the liquid and solid phases at equilibrium, and to approach the sorption mechanism involved. 

**Figure 4:**
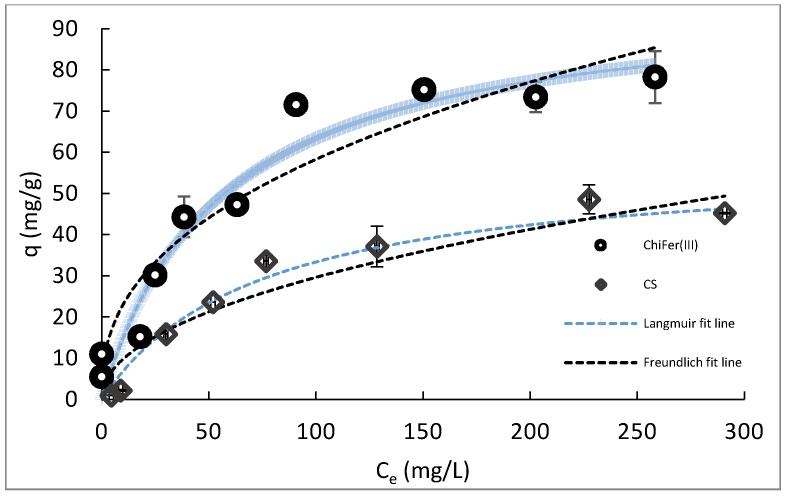
Adsorption isotherm. (T = 20 °C; pH: 6, dose: 1 g/L, contact time: 24 h, agitation rate: 150 rpm).

Figure 4 shows the resulting isotherms of CS and ChiFer(III), adjusted to the Langmuir and Freundlich non-linear models.

Figure 4 reports on the improvement of performance towards Sb(III) with the introduction of iron(III) into the chitosan matrix. The ChiFer(III) material showed a better sorption capacity in comparison with neat chitosan; this was because the incorporation of iron resulted in the enhancement of the material towards the Sb(III) sorption. The mathematical adjustment was done by using the Langmuir and Freundlich non-linear models. Langmuir fit better than the Freundlich model, resulting in a maximum uptake capacity (q_max_) of 98.68 ± 9.81 mg/g. According to the Langmuir theory, the formation of a monolayer between the adsorbate and adsorbent is assumed [38], which could be the adsorption phenomena that are predominant in this study. The results could be compared with [18,19], in which the incorporation of iron on chitosan improved the sorption of Nd(III) and Hg(II), besides, in comparison with other biosorbents, the performance of ChiFer(III) material is very competitive in terms of q_max_; for example, [39] *Sargassum* sp., which can adsorb 18.1 mg of antimonite per gram of biosorbent. In another case, [13] nano-titania chitosan composite was used, and this was highly efficient for Sb(III) adsorption, with a q_max_ of 84.91 mg/g Sb(III) and 22.61 mg/g of Sb(V). Although biosorbents based on chitosan matrix have been developed to remove several heavy metal elements, few chitosan-based biopolymers have been assessed for Sb(III) remediation. Table 1 shows a comparison between the different biomaterials proposed for Sb(III) removal from aqueous solutions.

### 3.4. Kinetics

**Figure 5:**
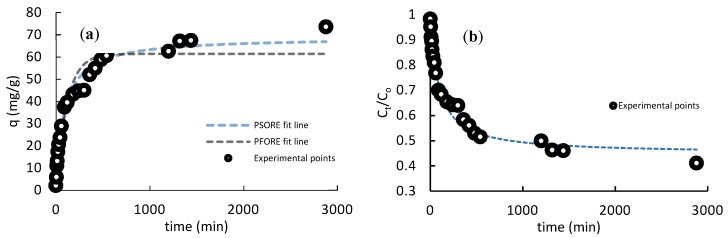
Kinetics isotherms. (T = 20 °C; pH 6, dose: 1 g/L, contact time: 48 h, agitation rate: 150 rpm).

The establishment of the rate law of the adsorbate–adsorbent through the kinetics study is important for understanding the mechanisms by which the solute is accumulated onto the sorbent surface [42], and the parameters obtained are also useful for reactor design. Adsorption kinetics can be represented by plotting the uptake vs time as well as the residual concentration vs time. Figure 5 shows the kinetic profile of Sb(III) on the ChiFer(III) material.

Figure 5a,b plotted the effect of contact time on the Sb(III) adsorption behavior; Figure 4a represents the adsorption capacity vs time; meanwhile, Figure 5b shows the remaining Sb(III) concentration vs time. In both, three pseudo-steps can be considered [23]: (i) an initial rate of adsorption, which took about 1.5 h, with a yield of 70%, and an experimental adsorption capacity (q_exp_) of 37.45 mg/g; this fast first step is normally controlled by film diffusion (external diffusion), (ii) a second step that took around 16 h, reaching a performance of 46% with q_exp_ of 66 mg/g, whose mass transfer control could be attributed to pore diffusion (intraparticle diffusion) through liquid film layer into the pores, and a third step attained at 28 h with 42% of efficiency, and q_exp_ of 72 mg/g corresponding to surface reaction, which the metal uptake rate is much slower than in the preceding steps.

The overall mass transfer in this study seemed to be controlled by the three steps, and this can be explained by the concentration gradient reducing progressively over time, decreasing the dynamics between the active sites and sorbate molecules [35]; as a consequence, the adsorption rate was faster at the beginning, and it became slowed as the reaction continued. On the other hand, no linear models of PFORE and PSORE were assessed, and the PSORE (R^2^ = 0.97) fitted better than PFORE (R^2^ = 0.92) in terms of R^2^. A difference between PFORE and PSORE was that the PFORE assumed that the rate of occupation of the adsorption sites was proportional to the number of unoccupied sites; meanwhile, the PSORE model considers that the uptake rate is second-order with respect to the available surfaces sites [24]; that is to say that the rate of occupation of the adsorption sites was proportional to the square of the number of unoccupied sites. Moreover, the PSORE was based on the assumption that the rate-limiting step may be the chemisorption, involving the exchange of electrons between the biosorbent and the sorbate [43]. Similarly, some studies demonstrated a fit by PSORE in similar conditions, such as [18,19,41].

### 3.5. Ion Competition

**Figure 6:**
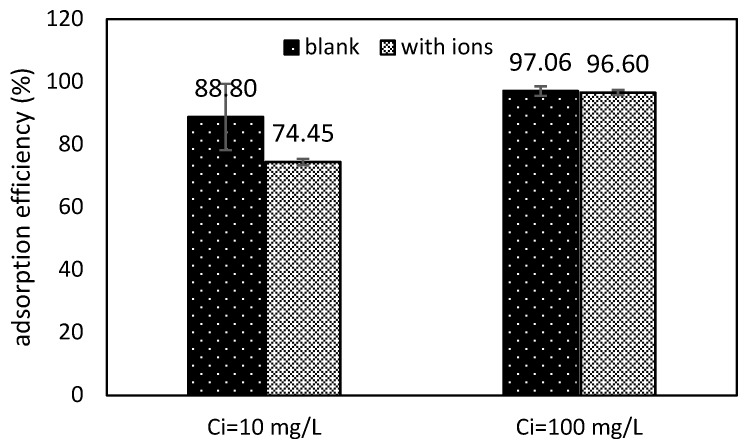
Ion competition (Sorbent dose: 1 g/L; contact time: 24 h; agitation rate: 150 rpm).

In real wastewater, many ions coexist, representing competitors during the adsorption process. To know the interaction and the competition of some ions, two synthetic wastewaters were prepared with Sb(III) concentrations of 10 mg/L and 100 mg/L; both solutions had the same concentrations of other ions (Na_2_NO_3_: 0.15 mM, NaCl:1 mM, Na_2_CO_3_: 2 mM Na_2_SO_4_: 2 mM). The choice of the salts and their concentrations attempted to simulate real surface water polluted by mining wastewater [44]. The results are shown in Figure 6.

The effect of the ion interaction is represented in Figure 6, which shows that the influence of ions in the adsorption process is more pronounced at low concentrations than at high Sb(III) concentration, decreasing the efficiency from 88% to 74% (with regard to cero salts) at an initial Sb(III) concentration of 10 mg/L, while at high concentrations, such as 100 mg Sb(III)/L, the influence of the ions was almost negligible, and the adsorption capacity decreased from 97.05 to 96.6%; however, at both concentrations, the differences were not statistically significant (Student’s *t*-test, α = 0.574). This phenomenon could be explained, as at a low concentration of Sb(III), the competition for active sites on sorbent material is more pronounced than at high Sb(III) concentration, where antimonite is predominant over the other salts. The presence of anions could reduce the adsorption efficiency, because of competition for interaction with active sites [45]. Some investigations have reported the great influence of some salts, which affects the adsorption capacity of the material; however, the material reported in this study is stable against the effects of the salts tested.

### 3.6. Desorption

**Figure 7:**
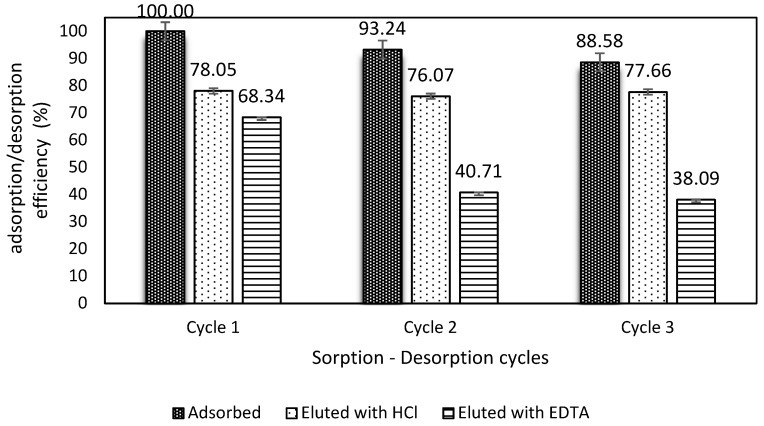
Adsorption–desorption cycles (Co = 50 mg/L, dose: 1 g/L, reaction time: 24 h, agitation: 150 rpm).

In order to assess the kind of eluent, as well as the reusability of the material over several adsorption–desorption cycles, two eluents (HCl pH 3.5 and EDTA 0.01M) for three cycles were evaluated. Figure 7 shows the behavior of the adsorption–desorption performance of the material.

It was noticed that HCl showed desorption recoveries of 78.05%, 76.07%, and 77.66% for cycles 1, 2, and 3, respectively, which resulted in a better desorption performance than EDTA. Conversely, EDTA could reach 68% at the first adsorption–desorption cycle, and then 40% and 38% for the second and third cycles, respectively. 

In recent studies carried out by our research department, this material showed good stability in terms of its performance of adsorption–desorption along several cycles. For instance, [18] tested various desorbents (i.e., HNO_3_, pH 3.5; HCl, pH 3.5; NaOH, pH 11; NaOH, pH 13; thiourea 0.1 M, pH 3.5; thiourea 0.05 M, pH 3.5; and EDTA 0.05 M, pH 10) to elute Hg(II) and Pb(II) from the ChiFer(III) material. There was showed that EDTA and HCl presented better desorption performances than the other eluents, with recoveries of up to 90% in a first screening; after that, just EDTA was used to determine the reusability, in which the recovery dropped to around 60–70% in the third cycle. Alternatively, in other studies, HCl pH 3.5 was successfully used (99.1% in the first adsorption–desorption cycle) to desorb boron and neodymium from a packed column filled with ChiFer(III) material for three cycles; however, during the third adsorption–desorption cycle, the desorption fell to 30.3% [19].

The differences in the elution performance of these two desorbent solutions could be related to the adsorption electrostatic forces or the chelating interactions between Sb(III) and ChiFer(III). EDTA is well known for its chelating properties, and in conjunction with the alkaline conditions, it remains deprotonated, which could favor an interchange between Sb(III) and the interchangeable groups of EDTA; however, it does not have a high desorption rate. On the other hand, HCl pH 3.5, which is high protonated, can remove Sb(III) more easily, probably due to the electrostatic interactions that are induced through the desorption process acting stronger than the chelating forces. Moreover, regarding the bead stability at pH 3.5, the ChiFer(III) remained in good shape during the experimentation; however, under pH 3, the material suffered progressive degradation (Supplementary Materials, Figure S6).

## 4. Conclusions

The present study showed a polysaccharide-based material with a high adsorption capacity of one of the most toxic elements, such as Sb(III). The biomaterial was obtained by a simple blending of chitosan and iron(III) prior to simultaneous bed formation and coagulation. The material was characterized by several techniques, which helped to elucidate the mechanism involved in governing adsorption. The applicability of the material towards the process scale-up was evaluated through a pH study, the effect of initial concentration, kinetics, ion effects, and adsorption–desorption cycles. It was found that the incorporation of iron(III) into the chitosan matrix improved the material adsorption capacity by around 50%. The mechanism involved in the adsorption corresponded mainly to chemisorption, and at minor scale, to electrostatic attraction. The Langmuir model fitted the equilibrium isotherms better, while PSORE fitted with major correlation to the kinetics phenomena. The ChiFer(III) biomaterial could be used to remove Sb(III) from water in the presence of some ions different to Sb(III), and it can be reused by for to three adsorption–desorption cycles.

## Figures and Tables

**Table 1:** Adsorption capacity of Sb(III) with different biomaterials.

Material	pH	T	q_max_	Isotherm Fitting	Reference
		(°C)	(mg/g)		
*Sargassum* sp.			18.1		[39]
Nano-titania chitosan—crosslinking with epichlorohydrin			84.91		[40]
Raw pumice	5	20	44.80		[14]
Chitosan-modified pumice	5	20	88.90		[14]
Green bean husk	4	25	20.14		[41]
ChiFer(III) beads (present study)	6	room temperature	98.68	Langmuir	
Neat chitosan (present study)	6	room temperature	57.99	Langmuir	

## Supplementary Materials

The following are available online at http://www.mdpi.com/2073-4360/11/2/351/s1, Figure S1: FTIR spectra of neat chitosan—CS. Figure S2. FTIR spectra of ChiFer(III) before Sb(III) adsorption. Figure S3. FTIR spectra of ChiFer(III) after Sb(III) adsorption. Figure S4. ChiFer(III) beads at time = 0. Figure S5. ChiFer(III) beads at time = 24 h. Figure S6. ChiFer(III) beads at time = 48 h. Figure S7. Particle size of ChiFer(III) material.

## Abbreviations

CS	neat chitosan powder
ChiFer(III)	chitosan/iron(III) hydroxide beads
