# Supplementary material for: Antimony Removal from Water by a Chitosan-Iron(III)[ChiFer(III)] Biocomposite
Source: Polymers (Basel). 2019 Feb 18;11(2):351. doi: 10.3390/polym11020351 (PMC6419170; doi:10.3390/polym11020351)

Supplementary Materials

Figure S1. FTIR spectra of neat chitosan - CS

Figure S2. FTIR spectra of ChiFer(III) before Sb(III) adsorption

Figure S3. FTIR spectra of ChiFer(III) after Sb(III) adsorption

Figure S4. ChiFer(III) beads at the time=0

Figure S5. ChiFer(III) beads at the time=24 h

Figure S6. ChiFer(III) beads at the time=48 h

Figure S7. Particle size of ChiFer(III) material

Figure S1. FTIR spectra of neat chitosan - CS

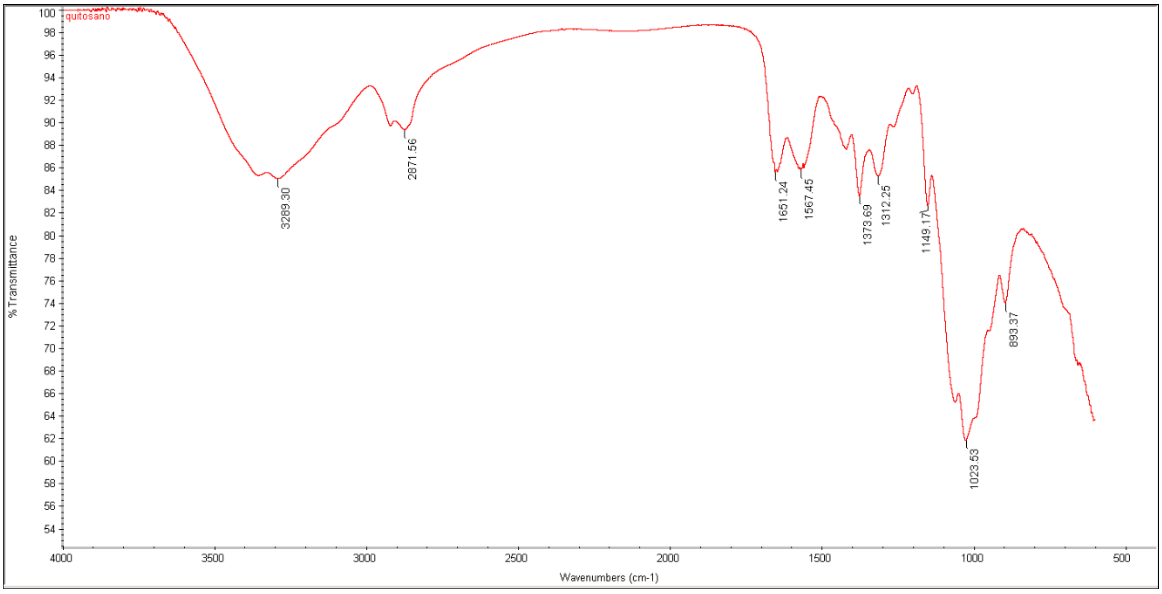

Figure S2. FTIR spectra of ChiFer(III) before Sb(III) adsorption

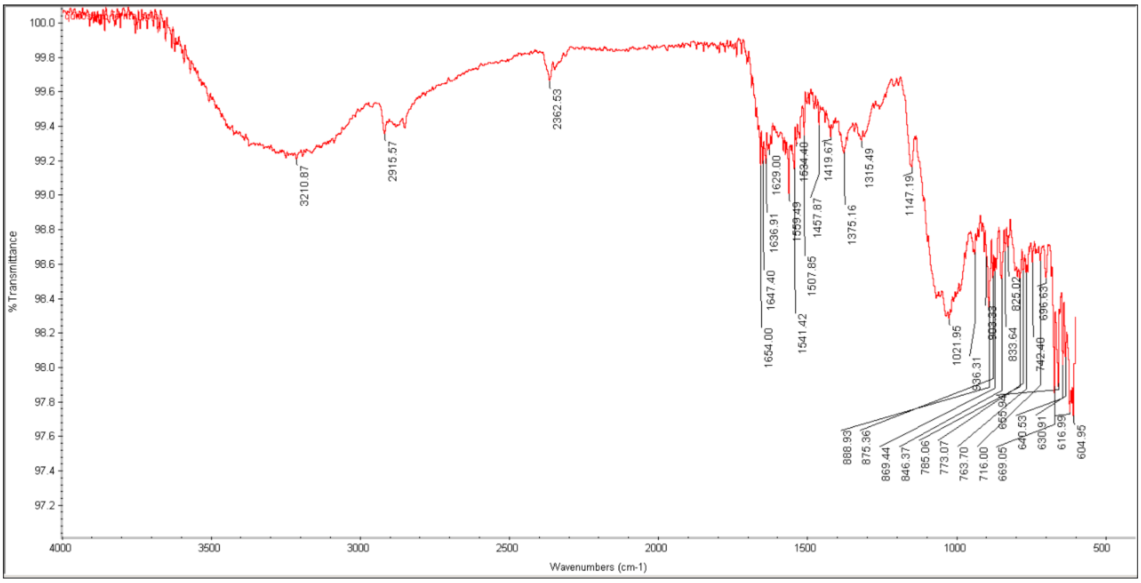

Figure S3. FTIR spectra of ChiFer(III) after Sb(III) adsorption

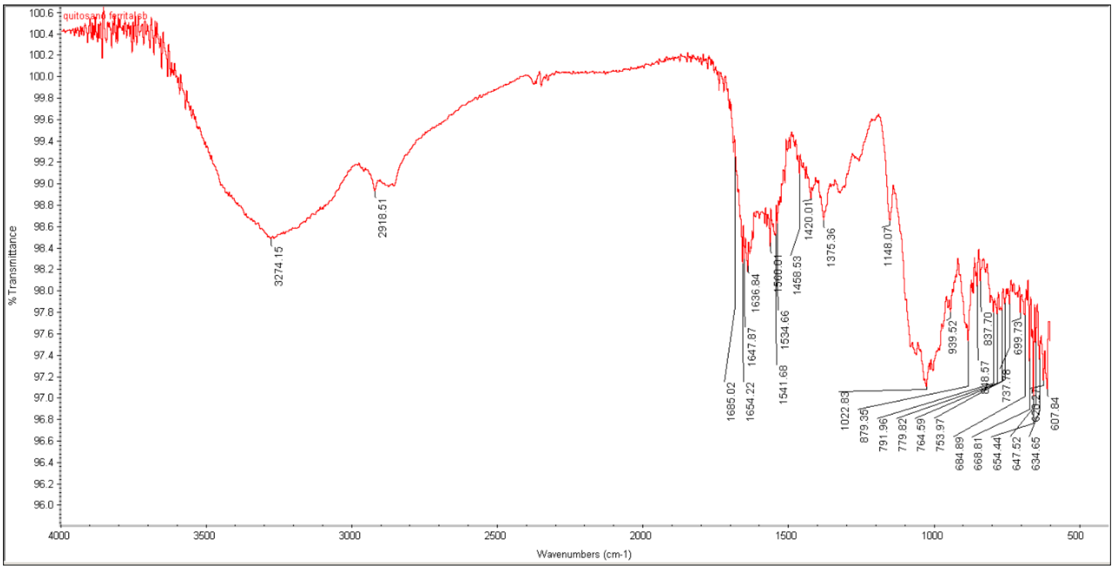

ESTABILITY OF THE ChiFer(III) material

Figure S4. ChiFer(III) beads at the time=0

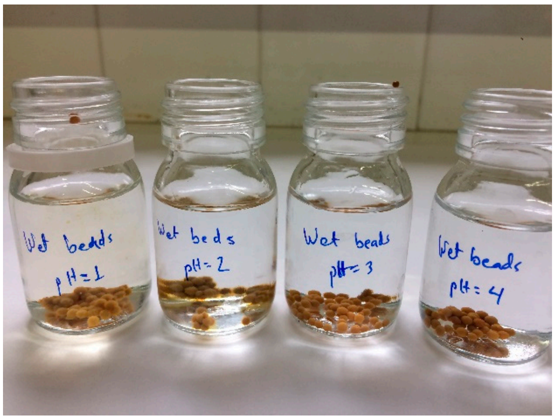

Figure S5. ChiFer(III) beads at the time=24 h

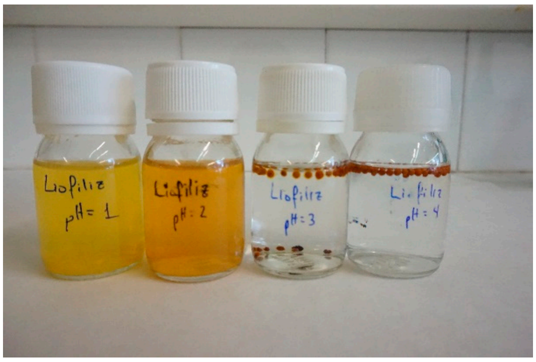

Figure S6. ChiFer(III) beads at the time=48 h

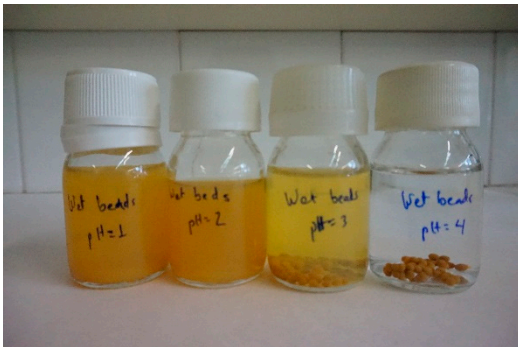

Figure S7. Particle size of ChiFer(III) material

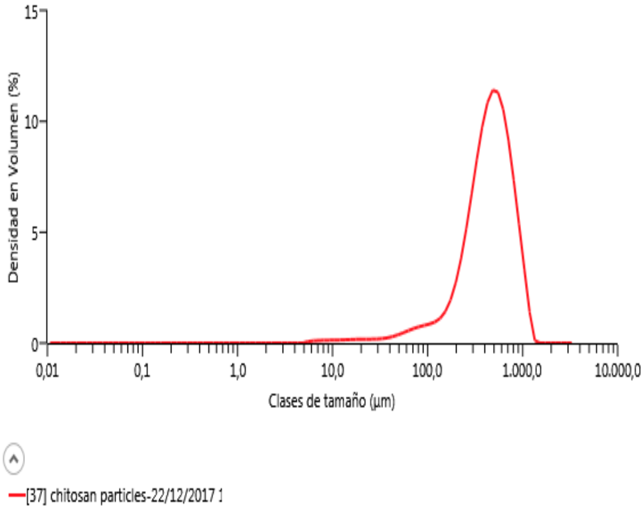

Supplement: Supplementary file 1 [file polymers-11-00351-s001.pdf]
